# Lifetime Prediction and Aging Characteristics of HTV-SiR Under Coupled Electro–Thermo–Hygro–Mechanical Stresses

**DOI:** 10.3390/polym18080955

**Published:** 2026-04-14

**Authors:** Ben Shang, Wenjie Fu, Lei Yang, Qifan Yang, Zian Yuan, Zijiang Wang, Youping Fan

**Affiliations:** 1 School of Electrical Engineering and Automation, Wuhan University, Wuhan 430072, China; 2015302540235@whu.edu.cn (B.S.); 2021302191333@whu.edu.cn (W.F.); 2 Institute of Next Generation Power Systems and International Standards, Wuhan University, Wuhan 430072, China; qfyang@whu.edu.cn (Q.Y.); 2024106560083@whu.edu.cn (Z.Y.); 3 China Electric Power Research Institute, Wuhan 430072, China; yanglei@163.com; 4 School of Electrical Engineering, North China University of Water Resources and Electric Power, Wuhan 430072, China

**Keywords:** composite insulator, silicone rubber, generalized Eyring relationship, lifetime prediction, polymer degradation

## Abstract

To investigate the aging behavior of high-temperature-vulcanized silicone rubber (HTV-SiR) used in composite insulator sheds under coupled electrical, thermal, humidity, and mechanical stresses, accelerated aging tests were conducted to emulate the service conditions of ±800 kV ultra-high-voltage direct current (UHVDC) systems in Guangzhou, China. The physicochemical, mechanical, and electrical properties of the specimens were systematically characterized. The results show simultaneous degradation of both electrical and mechanical performance. In particular, the tensile strength exhibits a significant monotonic decrease and drops to 49.52% of its initial value under the most severe condition (0.5 kV·mm^−1^ and 5% tensile strain) after 75 days. In contrast, the DC breakdown strength shows a non-monotonic “rise-then-fall” trend and decreases more markedly with increasing tensile strain. To address the one-shot and destructive nature of tensile testing and the associated statistical uncertainties, a lifetime prediction framework was developed by integrating a generalized Eyring acceleration relation with a stochastic degradation process. Under representative service conditions of 0.09 kV·mm^−1^ and 0.2% tensile strain, the predicted lifetimes corresponding to failure probabilities of 10%, 75%, and 90% are 1.77, 9.08, and 17.90 years, respectively. The applicability of the model is supported by field-aged specimens. These findings provide a mechanistically grounded and reliability-oriented basis for condition assessment, lifetime-margin evaluation, material screening, and maintenance planning of UHVDC composite insulators operating in hot–humid environments.

## 1. Introduction

High-temperature-vulcanized silicone rubber (HTV-SiR) is the dominant housing and shed material for composite insulators because it combines hydrophobicity, elasticity, low density, and satisfactory electrical insulation performance [1,2]. However, prolonged outdoor service gradually degrades HTV-SiR through cracking, chalking, hardening, hydrophobicity loss, and mechanical embrittlement, which can compromise both the surface insulation function and the protection of the glass-fiber-reinforced core rod [3,4]. Consequently, an in-depth understanding of the degradation behavior of HTV-SiR, together with reliable assessment of its service life, is essential for the reliable operation of UHVDC composite insulators [1,2].

In actual service, HTV-SiR is subjected to multiple stresses. The shed surface is simultaneously exposed to electrical, thermal, and humidity stresses, and the housing bears sustained mechanical loading transferred from the core rod and fittings. This situation is especially critical for UHVDC tension insulators, in which long-term axial load can introduce persistent tensile deformation in the HTV-SiR housing and thereby alter defect evolution and accelerate material deterioration [5,6,7,8].

Previous studies have clarified several key aspects of HTV-SiR aging. Hygrothermal and thermo-oxidative studies showed that hydrolysis, chain scission, re-crosslinking, and filler precipitation jointly control the evolution of chemical structure and macroscopic properties, and that the interaction between temperature and humidity significantly affects degradation kinetics [3,4]. Thermo–mechanical investigations further demonstrated that tensile stress can hinder the further development of the crosslinked network and accelerate the loss of mechanical and electrical performance by promoting molecular-chain alignment, interfacial debonding, and crack growth [5,8]. In addition, recent multi-factor studies involving electric field, humidity, temperature cycling, and other environmental factors confirmed that electrical stress can substantially change the degradation pathway and therefore should not be neglected in accelerated aging evaluation of silicone rubber used in composite insulators [9,10].

Despite this progress, the available literature still has clear limitations. First, most reported studies remain confined to hygrothermal aging, thermo–mechanical aging, or electric–environmental coupling, and existing thermo–mechanical studies have mainly focused on cable accessories [3,5,8,9,10]. Nevertheless, electric field, temperature, humidity, and sustained tensile strain collectively represent the actual service conditions of HTV-SiR sheds in UHVDC tension strings, and their simultaneous action has rarely been investigated within a unified framework [8,9,10]. Second, although several accelerated lifetime approaches have been reported for rubber materials and polymeric insulation, including Arrhenius-based, Eyring-based, and time–temperature or humidity-acceleration-based methods [3,11,12], HTV-SiR-specific studies have mainly focused on thermal or heat–moisture environments. For example, Cheng et al. proposed lifespan evaluation approaches for composite insulators and silicone rubber materials in hot and humid regions, and Zhang et al. recently established a generalized Eyring-based model for HTV-SiR under hygrothermal aging [3,13,14]. However, these models do not explicitly quantify the additional acceleration introduced by electric field and sustained tensile strain.

A further limitation concerns the statistical nature of the degradation data. Tensile strength measurements are obtained from destructive end-point tests on different specimens rather than from continuous monitoring of the same sample. Therefore, specimen-to-specimen dispersion, uncertainty in the initial state, and stochastic fluctuations during degradation should be explicitly incorporated into lifetime prediction, especially when the sample size is limited [15,16,17]. A purely deterministic degradation trajectory is insufficient to represent this type of data and may lead to biased lifetime estimates.

Against this background, the present study investigates the aging behavior of HTV-SiR under coupled electro–thermo–hygro–mechanical stresses using service-relevant conditions for UHVDC composite tension insulators. The novelty of this study lies in three aspects. First, the accelerated aging framework explicitly combines electric field and sustained tensile strain with heat and humidity, rather than considering only heat–moisture or thermo–mechanical coupling [8,15]. Second, the evolution of mechanical, electrical, and microstructural properties is characterized and analyzed jointly, providing a clearer mechanistic basis for interpreting multi-stress degradation [3,8,15]. Third, a generalized Eyring acceleration relation is integrated with a stochastic degradation process to accommodate destructive tensile data and to enable reliability-oriented, quantile-based lifetime prediction. Therefore, this study aims not only to clarify the coupled aging characteristics of HTV-SiR, but also to provide a more statistically appropriate framework for lifetime assessment of composite insulators in complex service environments.

## 2. Accelerated Aging Test

### 2.1. Test Specimen Preparation

The properties of HTV-SiR are strongly influenced by material formulation and processing conditions. To ensure that the subsequent validation of the lifetime prediction model is representative of field operating conditions, the HTV-SiR used in this study was taken from a spare composite insulator supplied by China Southern Power Grid UHV Transmission Co. The composite insulator (type: FXBZ ±800/530B) was manufactured in 2009, and insulators with the same formulation and process have been in service for approximately 15 years on a ±800 kV UHVDC transmission line in Guangzhou.

**Figure 1:**
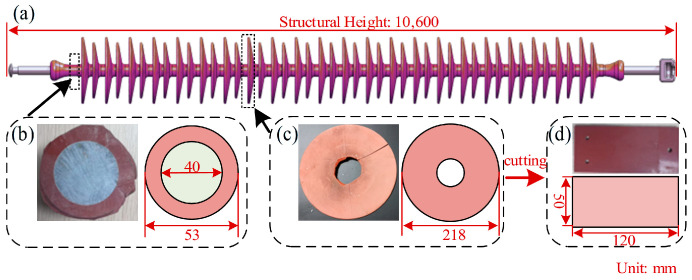
Schematic diagram of the sample preparation. (**a**) The FXBZ ±800/530B composite insulator sample. (**b**) Diametral sections of insulator. (**c**) Composite shed. (**d**) HTV-SiR specimen.

The sheds were cut into plates measuring 150 mm × 150 mm × 2 mm using a hydraulic press and were then used as test specimens in the aging tests, as shown in Figure 1. The constituents and basic properties of the specimens are summarized in Table 1.

### 2.2. Accelerated Test Design

#### 2.2.1. Selection of Experimental Parameters

The aging test conditions were designed based on climatic statistics and operational data from a ±800 kV UHVDC transmission project in Guangzhou. Meteorological records from 2009 to 2024 show that Guangzhou experiences a historical maximum temperature of 39.3 °C and a maximum monthly mean relative humidity of 86%. The Chinese national standard GB/T 19519-2014 [18] specifies a heating temperature of 50 ± 5 °C for thermal–mechanical testing of insulators. To balance engineering relevance with accelerated aging effects, this study established test conditions at 50 °C/85% RH.

Literature [16,17] demonstrates that the surface electric intensity on composite insulators in this UHVDC project ranges from 0.050 to 0.375 kV·mm^−1^, with an average value of approximately 0.09 kV·mm^−1^. According to Chinese UHVDC design specifications, the maximum allowable electric intensity on the composite insulator surface is 0.50 kV·mm^−1^. Accordingly, two surface electric intensity levels of 0.30 and 0.50 kV·mm^−1^ were selected, representing approximately 3.3 and 5.6 times the average operating field strength, respectively.

**Figure 2:**
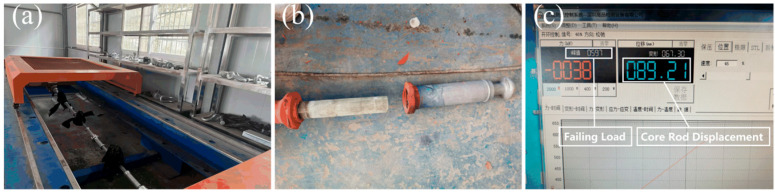
Tensile test of tensile composite insulators. (**a**) Appearance. (**b**) Failure mode. (**c**) Core rod displacement.

Mechanical stresses acting on the HTV-SiR sheds primarily arise from axial tension and bending-induced buckling loads. Literature [19] reports that under extreme bending conditions, the maximum tensile strain on the outer surface of the housing can reach 1.25%. To replicate the mechanical loading experienced by tension-type composite insulators in UHVDC service, a tensile failure test was conducted on an FXBZ ±800/530B insulator, as shown in Figure 2. The test recorded a maximum core rod displacement of 89.21 mm with a failing load of 597.0 kN, corresponding to a housing tensile strain of approximately 0.8%. Thus, tensile strain levels of 0%, 3%, and 5% were selected for this study.

Aging tests were conducted under all combinations of these electrical and mechanical loading conditions at 50 °C/85% RH for different durations, as summarized in Table 2.

#### 2.2.2. Testing Platform

**Figure 3:**
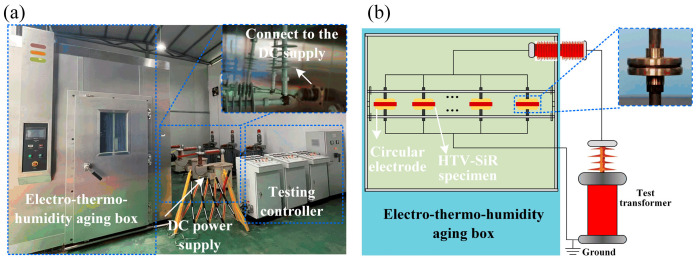
Electro–thermos–humidity aging box. (**a**) Appearance. (**b**) Schematic diagram.

**Figure 4:**
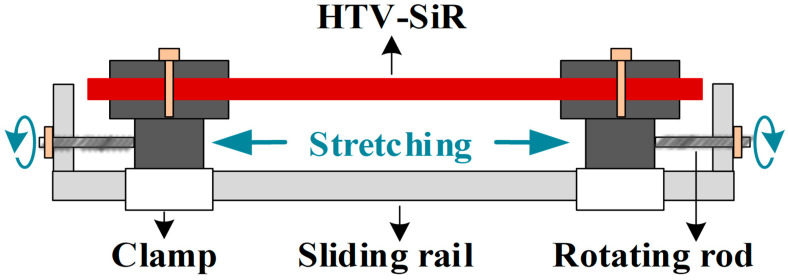
Tensile loading device.

An electro–thermo–humidity aging box was constructed, as shown in Figure 3. The temperature inside the chamber could be controlled from −40 °C to 150 °C, and the relative humidity from 20% to 98% RH. The temperature fluctuation is within ±0.5 °C, and the humidity fluctuation is within +2/−3% RH. The box was equipped with high-voltage bushings that allow connection to a DC high-voltage power supply, and circular brass electrodes with a diameter of 50 mm were used to simulate the electrical aging environment of HTV-SiR. In addition, a tensile loading device was designed, as shown in Figure 4. The applied tensile strain of the specimen could be adjusted by rotating the epoxy resin rotating rods located on both sides of the clamps.

### 2.3. Characterization and Measurement

1. Microstructural characterization (FTIR and SEM): FTIR spectra were acquired using an NICOLET 5700 spectrometer in attenuated total reflection (ATR) mode over the wavenumber range 400–4000 cm^−1^, with 32 scans collected for each specimen. SEM observations were performed using a TESCAN VEGA Compact scanning electron microscope to examine the surface morphology of the specimens after aging. Prior to imaging, the specimens were sputter-coated with a ~10 nm gold layer to improve surface conductivity. To ensure comparability, all images were collected from corresponding central surface regions of the specimens under identical sample preparation, magnification, and imaging conditions.
2. Crosslinking density testing: The crosslinking density was determined by the equilibrium swelling method in accordance with ASTM D2765-16 [20]. Approximately 0.20 g of each specimen was immersed in toluene and sealed for 24 h at 25 °C. After swelling, the specimens were gently blotted with filter paper to remove excess surface solvent and immediately weighed. The crosslinking density D was calculated as:

(1) $$D=1/2M_{c},M_{c}=-\rho_{0}V_{0}\sqrt[{3}]{\varphi }/\ln(1-\varphi )+\varphi +\lambda \varphi^{2}$$

where $M_{c}$ is the average molecular weight between crosslinks, φ is the polymer volume fraction in the swollen state, and $\rho_{0}$ is the density of HTV-SiR before swelling, $V_{0}=107\;\mathrm{ml}\cdot {\mathrm{mol}}^{-1}$, λ=0.465.

3. Shore hardness testing: Shore A hardness was measured using a Shore A durometer. The indenter was applied vertically and steadily to the material surface, and stabilized readings were recorded as the hardness values. Measurements were taken at points separated by at least 6 mm, with five readings averaged for each test point to ensure accuracy.
4. Contact angle testing: Static contact angles of specimens subjected to different aging conditions were measured using a Kino CAST 3.0 contact angle goniometer. During the test, approximately 5 μL of deionized water was dispensed onto the specimen surface with a micropipette.
5. Tensile strength testing: The tensile and tear strength of silicone rubber sheds were evaluated using an HP-1K tensile testing machine. Tensile strength was determined in accordance with ISO 527-1:2019 [21], using dumbbell-shaped specimens with a gauge length of 25 mm and a constant tensile rate of 500 mm·min^−1^.
6. DC dielectric properties testing: A Novocontrol GmbH Concept 40 broadband dielectric spectrometer was used to measure the DC dielectric properties of specimens at different aging states. The specimens were square plates with dimensions of 50 mm × 50 mm × 1 mm. The measurement temperature and frequency were set to 25 °C and 0.1 Hz, respectively, the applied voltage was 1 V. Before testing, circular gold electrodes with a diameter of 20 mm were sprayed onto the central area of both faces of each specimen.
7. DC breakdown strength testing: The DC breakdown strength was measured using a sphere–plate electrode configuration in accordance with GB/T 1408.1-2016 [22]. The measurement temperature was set to 25 °C. The applied voltage ramp rate was 1 kV/s. Before testing, the specimens were dried to minimize the influence of moisture. For each specimen, at least 15 valid breakdown values were obtained, and the results were analyzed using a Weibull distribution.
8. DC conductivity testing: DC volume conductivity was measured using an EST121 precision electrometer system in accordance with ASTM D257-14 [23]. A DC bias voltage of 500 V was applied, while the temperature was controlled at 25 ± 1 °C and the relative humidity at 70 ± 5% RH. The specimens were square plates with the same dimensions as those used in the DC dielectric property tests. The volumetric conductivity was calculated as:

(2) σ=d/R⋅A

where R is the stabilized resistance reading, d is the nominal specimen thickness, A is the controlled electrode contact area.

## 3. Testing Results Analyses

### 3.1. Chemical Performance Analysis

#### 3.1.1. Microstructural Characterization

**Figure 5:**
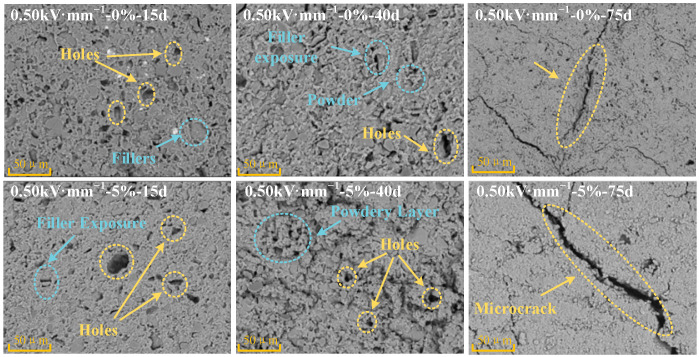
Representative SEM morphologies of the corresponding central surface regions of specimens under different aging conditions.

Figure 5 shows representative SEM morphologies of the corresponding central surface regions of specimens aged under the “0.5 kV·mm^−1^-0% tensile” and “0.5 kV·mm^−1^-5% tensile” conditions, allowing comparison of the dominant surface degradation features. The surface of the unaged specimen was dense and smooth, without visible defects or pores. For the specimen aged without tensile stress (0%), micrometer-scale pores were observed on the surface after 15 d of aging, accompanied by localized exposure and exudation of inorganic fillers. By 40 d of aging, the pore size had increased noticeably, and a loose, white powdery layer had formed on the surface. After 75 d of aging, microcracks developed across the surface, together with extensive accumulation of powdery residues. In contrast, the specimen subjected to 5% tensile strain exhibited more severe surface degradation after 75 d of combined aging. The number of surface cracks was markedly higher, and the cracks were longer and wider than those on the unstretched specimen.

This enhanced deterioration is attributed to the alignment of polymer chains along the stress direction under mechanical stretching, which promotes the formation of microvoids and stress concentrations at filler–matrix interfaces. Under the synergistic action of the electric field and the hygrothermal environment, these microstructural defects not only provide additional pathways for the penetration and diffusion of oxygen and water molecules but also induce local distortion of the electric field, which is consistent with accelerated backbone scission inferred from FTIR trends.

**Figure 6:**
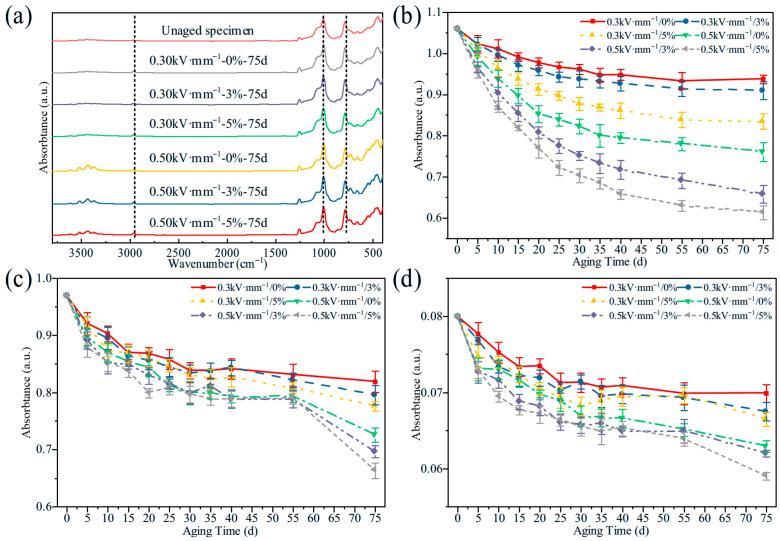
Changes in infrared spectral peak values of specimens under different aging conditions. (**a**) Infrared spectra of HTV-SiR. (**b**) Changes in Si-O-Si peak values. (**c**) Changes in Si-(CH_3_)_2_ peak values. (**d**) Changes in C-H peak values.

Figure 6 shows the evolution of surface functional groups during aging. No obvious new characteristic peaks were detected under the different aging conditions, indicating that aging mainly involved the consumption of existing functional groups. The characteristic peak intensities of Si–O–Si (1016 cm^−1^), Si–(CH_3_)_2_ (792 cm^−1^) and C–H (2964 cm^−1^) all decreased progressively with aging time, and the rate of decrease was initially rapid and then gradually slowed. After 75 days of aging, the mean Si–O–Si, Si–(CH_3_)_2_ and the C–H peaks of the “0.5 kV·mm^−1^-0% tensile” specimen had decreased by about 28.30%, 25.20% and 21.30% from their initial value, whereas those of the “0.3 kV·mm^−1^-5% tensile” specimen had decreased by 21.20%, 20.10% and 16.90% from their initial value. These results indicate that, within the ranges of electric field strength and tensile strain investigated in this study, increasing the electric field strength exerts a stronger promoting effect on degradation than tensile strain.

#### 3.1.2. Crosslinking Density Analysis

**Figure 7:**
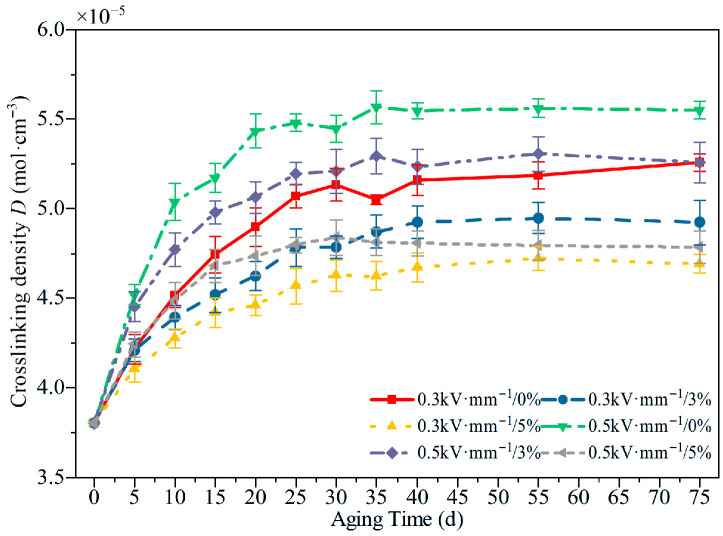
Crosslinking density of HTV-SiR specimens under different aging conditions.

Figure 7 shows the evolution of crosslink density under different aging conditions. The crosslink density of the unaged specimen was approximately 3.8 × 10^−5^ mol·cm^−3^. As the aging time increased, the crosslink density of the specimens generally increased at first and then tended to level off.

After 75 d of aging, the crosslink density remained higher than that of the unaged state. Under the same electric field, increasing tensile strain led to a pronounced reduction in the peak increment in crosslink density. At 0.5 kV·mm^−1^, the maximum observed crosslink density of the unstretched specimen reached 5.58 × 10^−5^ mol·cm^−3^, corresponding to an increase of about 46.8%, whereas the specimen aged under 5% tensile strain reached only 4.83 × 10^−5^ mol·cm^−3^, an increase of about 27.1%. As the electric field strength increased, both the growth rate and the magnitude of the crosslink density were markedly enhanced. For the unstretched specimen, the crosslink density under 0.3 kV·mm^−1^ continued to increase during the first 50 d of aging, whereas under 0.5 kV·mm^−1^, a distinct peak was observed at 35 d. These results indicated that, in the early stage of aging, oxidative crosslinking dominated the chemical reactions in silicone rubber, and with prolonged aging, the process gradually shifted toward main-chain scission. Tensile stress hindered the further development of the crosslinked network, whereas the electric field accelerated oxidative crosslinking in the hygrothermal environment. These changes observed by SEM and FTIR are consistent with the subsequent evolution of crosslink density. Specifically, the early formation of limited surface defects together with the moderate loss of characteristic groups, corresponds to an oxidative crosslinking-dominated stage, whereas the later development of pores, filler exposure, and cracks indicates the gradual transition toward chain scission and network deterioration.

### 3.2. Mechanical Properties Analysis

#### 3.2.1. Shore Hardness

**Figure 8:**
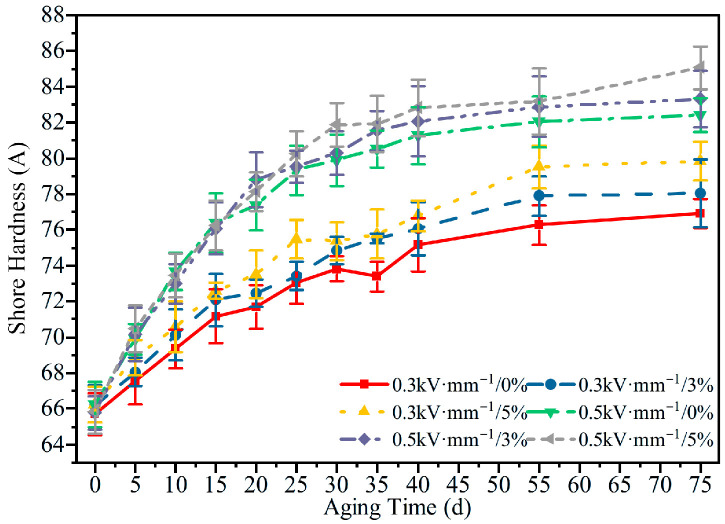
Shore hardness of HTV-SiR specimens under different aging conditions.

The hardness under different aging conditions is shown in Figure 8. As the aging time increased, the hardness of the specimens rose rapidly at first and then tended to level off. The electric field strength markedly accelerated the hardening process: under 5% tensile strain, after 75 d of aging, the average hardness at 0.5 kV·mm^−1^ was about 85.1 A, corresponding to an increase of 28.9% relative to the initial value of 66 A, whereas at 0.3 kV·mm^−1^, the hardness reached 79.9 A, an increase of 21.1%.

Although tensile stress also promoted hardness growth, its effect was relatively modest. Under 0.3 kV·mm^−1^ and 0.5 kV·mm^−1^, the average hardness of the unstretched specimens after 75 d was 76.9 and 82.4 A, respectively, only 3.0 and 2.7 A lower than those of the corresponding 5% tensile cases. These results further confirm that the electric field enhances the rigidity of the silicone rubber network by promoting oxidative crosslinking reactions, which manifests macroscopically as an increase in hardness.

#### 3.2.2. Contact Angle

**Figure 9:**
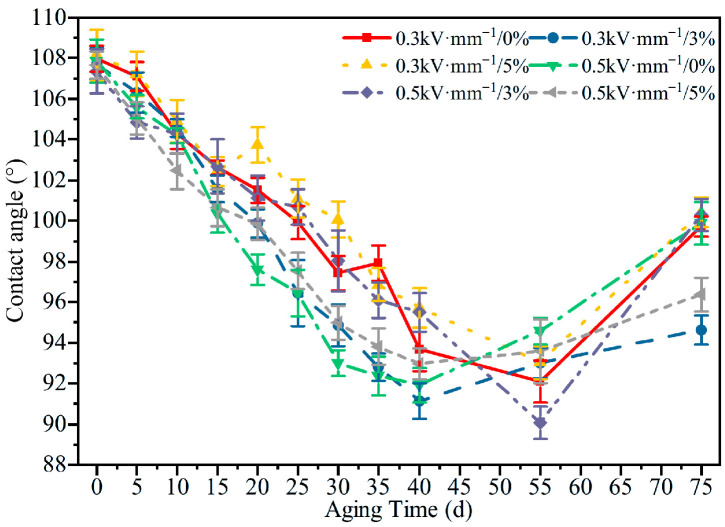
Contact angle of HTV-SiR specimens under different aging conditions.

The evolution of the contact angle under different aging conditions is shown in Figure 9. As the aging time increased, the surface contact angle of the specimens first decreased and then increased. Differences among the aging conditions were not pronounced, and after 75 d of aging most specimens still exhibited contact angles above 90°, indicating that even after severe combined aging, the HTV-SiR surface retained a certain degree of hydrophobicity.

This behavior can be explained by competing physicochemical processes at different aging stages. In the early stage, oxidation, crosslinking, and the migration of small molecules transform the outer surface into a more inorganic Si–O–rich layer, which increases the surface energy and enhances hydrophilicity, leading to a gradual decrease in the contact angle. In the later stage, however, a loose powdery layer progressively forms on the surface, and the accumulation of organic species such as polydimethylsiloxane (PDMS) partially restores and enhances the surface hydrophobicity, resulting in a subsequent increase in the contact angle.

#### 3.2.3. Tensile Strength

**Figure 10:**
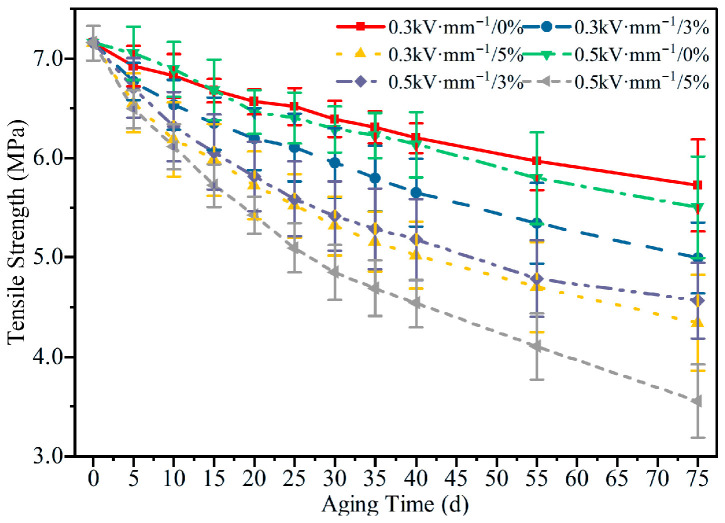
Tensile strength of HTV-SiR specimens under different aging conditions.

Figure 10 compares the evolution of tensile strength of HTV-SiR under different aging conditions. With increasing aging time, the tensile strength decreases markedly, and the degradation becomes more severe at higher tensile strain and higher electric field strength. After 75 d of aging, the tensile strengths under the six conditions are 80.01%, 69.01%, 60.39%, 74.98%, 63.23%, and 49.52% of the initial value (7.15 MPa), respectively. Comparison across aging conditions shows that both electric field strength and tensile stress accelerate the degradation of tensile properties, with the electric field exerting a more pronounced accelerating effect. Moreover, the two factors exhibit a clear synergistic effect in promoting accelerated aging. As aging proceeds, the crosslink density increases and progressively restricts chain mobility, so that the material becomes more brittle and the tensile strength continues to decrease. When the rates of chain scission and re-crosslinking approach a dynamic balance, the further reduction in tensile strength slows down and the curves tend to level off. It should be noted that the increase in hardness reflects network stiffening and reduced chain mobility, which increases brittleness and therefore accelerates the monotonic loss of tensile strength.

### 3.3. Electrical Properties Analysis

#### 3.3.1. DC Breakdown Strength

**Figure 11:**
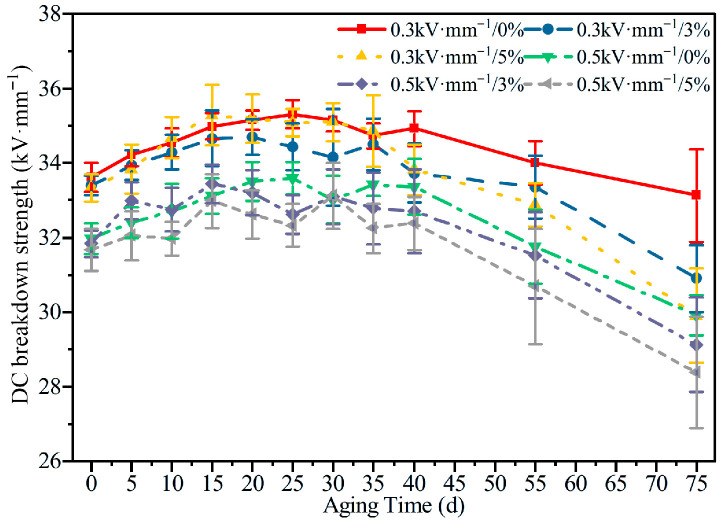
DC breakdown strength of HTV-SiR under different aging conditions.

Figure 11 compares the evolution of the DC breakdown strength of HTV-SiR under different aging conditions. With increasing aging time, the breakdown strength exhibits an initial rise followed by a subsequent decline, a trend that can be elucidated by the free volume breakdown theory.

In the early stage of aging, crosslinking reactions dominate, reducing the free volume of the rubber molecules and restricting electron acceleration, which leads to an increase in breakdown strength.

As aging proceeds, chain scission becomes more prevalent, resulting in an expansion of the free volume and an increase in the electron mean free path. This facilitates impact ionization and causes the breakdown strength to decrease significantly. After 75 d of aging under an electric field of 0.5 kV·mm^−1^, the average breakdown strengths for specimens with 0%, 3%, and 5% strain decrease by 6.44%, 8.51%, and 10.39% of their initial value, respectively.

#### 3.3.2. DC Dielectric Properties and Conductivity

**Figure 12:**
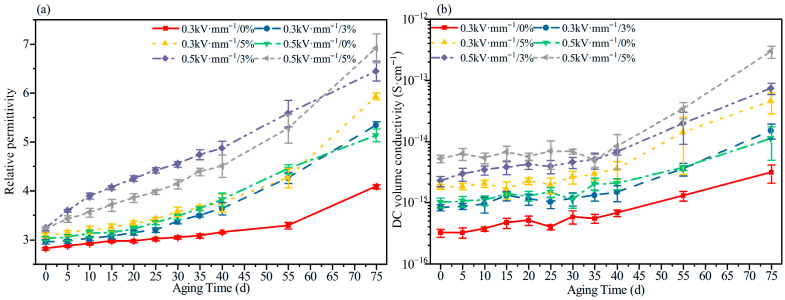
DC relative permittivity and volume conductivity of HTV-SiR under different aging conditions. (**a**) DC relative permittivity. (**b**) DC volume conductivity.

Figure 12 compares the evolution of the DC relative permittivity and DC volume conductivity of HTV-SiR under different aging conditions. With increasing aging time, both parameters exhibit a significant upward trend. Mechanistically, this is attributed to chain scission and oxidation reactions during the aging process, which lead to the accumulation of small molecular fragments and polar groups. These degradation byproducts enhance dipolar and interfacial polarization while simultaneously increasing carrier concentration and mobility, thereby driving the synchronized rise in the relative permittivity and volume conductivity. Furthermore, elevated electric field strength and tensile stress significantly amplify the growth magnitude of both parameters, demonstrating a distinct synergistic effect in accelerating the degradation of dielectric properties. Therefore, the monotonic increases in relative permittivity and DC conductivity should be interpreted together with the SEM-observed defect accumulation and the FTIR/crosslink density evidence of chain scission, rather than as isolated electrical responses.

### 3.4. Mechanism of Electro–Thermo–Hygro–Mechanical Aging

**Figure 13:**
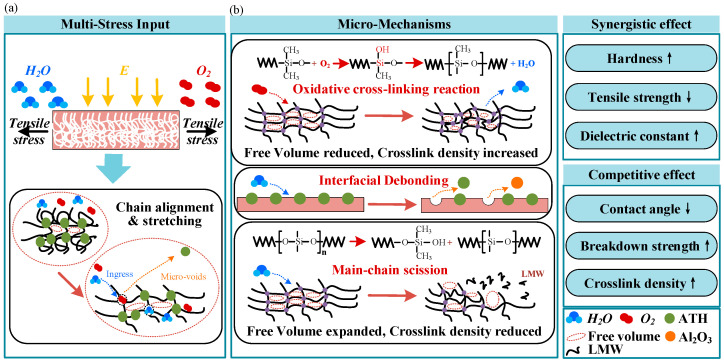
Electro–thermos–humidity–mechanical aging mechanism of HTV-SiR. (**a**) Multi-stress environmental inputs and macromolecular structural evolution; (**b**) Microscopic degradation mechanisms and their effects on material properties.

Based on the SEM observations, FTIR spectra, and crosslink density results, the aging mechanism of HTV-SiR under coupled electro–thermos–hygro–mechanical stresses can be interpreted as a transition from early oxidative crosslinking to later main-chain scission, as illustrated in Figure 13 [3,5]. Physically, as shown in Figure 13a, external tensile stress aligns and stretches the coiled polymer chains, generating stress concentration and microvoids at the filler–matrix interface [8]. These defects weaken interfacial bonding and provide additional pathways for the ingress of oxygen and moisture. Chemically, as shown in Figure 13b, the aging process proceeds through two dominant stages. In the early stage, oxidative crosslinking of side-chain methyl groups dominates, leading to densification of the molecular network and a reduction in free volume [15]. This interpretation is consistent with the increase in crosslink density from 3.8 × 10^−5^ mol·cm^−3^ in the unaged state to a maximum of 5.58 × 10^−5^ mol·cm^−3^ under 0.5 kV·mm^−1^ without tensile strain. With further aging, however, the degradation gradually shifts toward main-chain scission. Fracture of the Si-O-Si backbone generates low-molecular-weight fragments and polar groups, promotes filler exudation and crack development, and eventually causes collapse of the molecular network together with a pronounced increase in free volume [4]. Under the same electric field of 0.5 kV·mm^−1^, the maximum crosslink density under 5% tensile strain reaches only 4.83 × 10^−5^ mol·cm^−3^, indicating that tensile stress suppresses the further development of the crosslinked network and accelerates structural damage [15].

A comparison of Figure 5, Figure 6, Figure 7, Figure 8, Figure 9, Figure 10, Figure 11 and Figure 12 reveals a clear microstructure–property linkage for HTV-SiR under coupled aging stresses. In the early stage of aging, SEM shows only isolated pores and limited filler exposure, whereas FTIR already indicates progressive depletion of Si-O-Si, Si-(CH_3_)_2_, and C-H groups. This stage corresponds to network densification, which explains the increase in hardness from 66 A to about 85.1 A and also accounts for the temporary increase in DC breakdown strength because the denser network reduces free volume, enhances charge trapping, and suppresses carrier acceleration [5]. However, as aging proceeds, enlarged pores, severe filler exudation, and surface cracks become dominant, especially under tensile loading, indicating that chain scission and interfacial debonding gradually override the beneficial effect of early crosslinking [8]. As a result, the material becomes progressively harder but more brittle, so that the tensile strength decreases continuously and falls to 49.52% of its initial value under the most severe condition after 75 d [8]. Meanwhile, under 0.5 kV·mm^−1^, the DC breakdown strength decreases by 6.44%, 8.51%, and 10.39% for 0%, 3%, and 5% tensile strain, respectively, confirming that crack growth and free volume expansion ultimately govern the electrical failure behavior [5]. At the same time, the continuous increases in relative permittivity and DC conductivity are consistent with the accumulation of polar groups, low-molecular-weight fragments, and defect-assisted transport pathways generated by chain scission and interfacial debonding [5,15]. Therefore, the degradation of electrical and mechanical properties does not proceed independently; instead, both originate from the same coupled microstructural evolution of HTV-SiR [8].

## 4. Degradation Model and Lifetime Prediction

The selection of a characteristic parameter that reliably reflects the material’s aging state is critical for lifetime assessment. In this study, tensile strength is adopted as the degradation indicator because it exhibits a clearly monotonic decay and high sensitivity to aging severity over the entire service life, thereby reducing the modeling uncertainty associated with complex non-linear degradation behaviors.

### 4.1. Degradation Data Uncertainties Analysis

The exponential function is widely adopted to describe the degradation of the tensile strength of HTV-SiR. The general form is expressed as [24]:

(3) $$x(t)=x(0)\cdot \exp\left(-k\cdot t^{\rho }\right)$$

where x(t) is the degradation process of the HTV-SiR tensile strength, x(0) is the initial tensile strength, k is the chemical reaction factor, t is the accelerated aging time, and ρ is the shape parameter of the decay.

**Figure 14:**
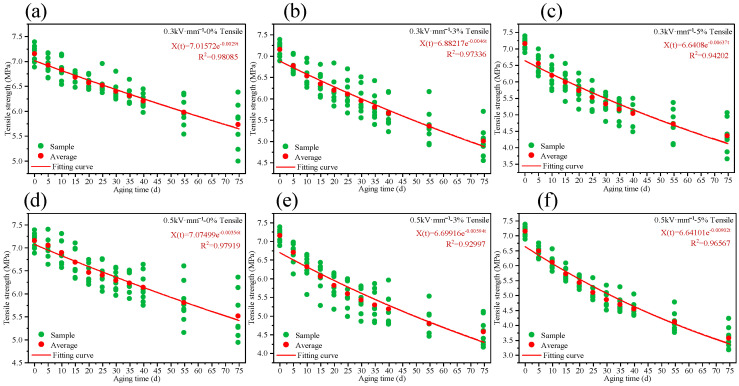
The distributions of tensile strength data under various aging conditions: (**a**) 0.3 kV·mm^−1^-0% tensile. (**b**) 0.3 kV·mm^−1^-3% tensile. (**c**) 0.3 kV·mm^−1^-5% tensile. (**d**) 0.5 kV·mm^−1^-0% tensile. (**e**) 0.5 kV·mm^−1^-3% tensile. (**f**) 0.5 kV·mm^−1^-5% tensile.

Equation (3) represents a deterministic degradation model, which assumes that the degradation of HTV-SiR is a continuous-time process observed on the same specimen. However, in tensile testing, the specimen is essentially a one-shot device: it performs its intended function only once and is destroyed immediately after the test. Differences in initial performance, fluctuations in aging conditions over long aging periods, and measurement noise all lead to pronounced dispersion in such destructive data. Figure 14 illustrates the distributions of tensile strength data under various aging conditions, with eight parallel specimens tested at each time point. It is evident that even under identical stress levels and aging durations, the specimens exhibit significant scatter in tensile strength, indicating intrinsic stochasticity in the degradation process.

The aging data were fitted using Equation (3), with results summarized in Table 3. For all aging conditions, the coefficient of determination $R^{2}$ exceeds 0.92, suggesting that model (3) reproduces the average degradation trend reasonably well. However, this deterministic approach reflects only the macroscopic mean behavior. It fails to account for the actual dispersion of the data. The scattered points often contain critical information on early failures; neglecting them may introduce substantial bias into lifetime prediction. Taking the “0.3 kV·mm^−1^-0% tensile” and “0.5 kV·mm^−1^-5% tensile” conditions as examples, at 75 d of aging, the minimum tensile strengths are approximately 4.98 MPa and 3.16 MPa, respectively. According to Equation (3), these strength levels correspond to predicted aging times of 117.14 d and 80.67 d, leading to relative errors of 56.18% and 7.56% with respect to the actual aging time. This indicates that defining lifetime solely based on point estimates from a deterministic model inevitably overlooks degradation randomness and tends to produce overly optimistic predictions that do not meet engineering reliability requirements.

Furthermore, comparison of the prediction errors reveals that this bias decreases as the electric field strength and tensile strain increase. Physically, both electric field and tensile strain accelerate the mechanical degradation of the material. Under high-stress conditions, the degradation trajectories of individual specimens tend to become more uniform, thereby attenuating the random fluctuations caused by microstructural heterogeneity and initial defect variability. In contrast, under mild operating conditions, stochastic factors have a more pronounced influence on the failure time. Given that the actual service environment typically corresponds to moderate or low stress levels, this further underscores the necessity of employing a stochastic degradation model in lifetime prediction to properly account for such uncertainty.

### 4.2. Multi-Stress Coupled Accelerated Model

To address the limitations of deterministic modeling and to simultaneously account for the uncertainty in initial performance and the intrinsic randomness of the aging process, this study introduces a Wiener process [25] to model the degradation path. The stochastic model is expressed as follows:

(4) $$X(t)=X(0)-k\int_{0}^{t}\Lambda (\theta )d\theta +\delta B(t)$$

where Λ(θ) is a time-scale function and, according to Equation (3), satisfies $\int_{0}^{t}\Lambda (\theta )d\theta =t^{\rho }$. The initial state X(0)=ln(x(0)) follows a distribution with mean m and variance $\sigma^{2}$, reflecting the average initial tensile strength of HTV-SiR and the specimen-to-specimen variability caused by manufacturing heterogeneity and pre-existing defects; σ is the diffusion coefficient; B(t) is a Brownian motion, and σB(t) captures the uncertainties arising from aging and measurement noise.

The chemical reaction factor k in Equation (4) is governed by the underlying physicochemical reaction kinetics. For HTV-SiR subjected to hygrothermal aging, the reaction factor is typically described by combining the generalized Eyring model and the Arrhenius equation [3]:

(5) $$k(H,T)=\alpha \cdot \exp\left\{-\frac{R_{a}}{K_{B}T}-\frac{\beta_{h}}{H}-\frac{\mu_{H,T}}{H\cdot T}\right\}$$

where α is the aging coefficient, $R_{a}$ is the activation energy of the material, $K_{B}$ is the Boltzmann constant, T is the absolute temperature, H denotes humidity, $\beta_{h}$ is the humidity aging index, and $\mu_{H,T}$ is the hygrothermal coupling coefficient.

In this study, we investigate the combined effects of electric field and tensile stress alongside hygrothermal conditions. Accelerated aging tests and prior studies [9] revealed that these four factors exhibit a pronounced synergistic interaction that significantly accelerates the mechanical degradation of HTV-SiR. Empirical evidence further indicates that the individual acceleration effects of electric field strength and tensile stress follow inverse power–law relationships [10]. Building on this basis, we extend Equation (5) to incorporate not only their independent contributions but also their mutual coupling. The resulting multi-stress coupling acceleration factor model is given by:

(6) $$k(E,M,H,T)=\alpha \cdot E^{-\beta_{e}}\cdot M^{-\beta_{m}}\cdot \exp\left\{-\frac{R_{a}}{K_{B}T}-\frac{\beta_{h}}{H}-\frac{\mu }{H\cdot T\cdot E\cdot M}\right\}$$

where $\beta_{e}$, $\beta_{m}$ denote the aging indices associated with the individual effects of electric field strength E and tensile strain M, respectively, and μ characterizes the coupling among the four stress factors.

In summary, by substituting the acceleration factor Equation (6) into the stochastic framework Equation (4), the complete degradation model for the tensile strength of HTV-SiR developed in this study is expressed as:

(7) $$\left\{\begin{array}{l} X(t)=X(0)-k\cdot t^{\rho }+\delta B(t)\\ k=\alpha \cdot E^{-\beta_{e}}\cdot M^{-\beta_{m}}\cdot \exp\left\{-\frac{R_{a}}{K_{B}T}-\frac{\beta_{h}}{H}-\frac{\mu }{H\cdot T\cdot E\cdot M}\right\}\\ X(0)\sim D\left(m,\sigma^{2}\right)\;\mathrm{and}\;\mathrm{independent}\;\mathrm{of}\;\mathrm{each}\;\mathrm{other} \end{array}\right.$$

### 4.3. Parameter Estimation

In Equation (7), $K_{B}$ = 1.381 × 10^−23^ J·K^−1^ represents the Boltzmann constant. The full parameter vector is defined as $\theta =\{\alpha ,\rho ,m,\sigma^{2},R_{a},\beta_{h},\beta_{e},\beta_{m},\mu ,\delta^{2}\}$. It should be noted that the selected hygrothermal baseline of 50 °C/85% RH was established from field climatic statistics and relevant standards to represent a typical hot–humid operating environment for UHVDC composite tension insulators. However, because the present experimental design includes only one temperature–humidity combination, the hygrothermal kinetic parameters $R_{a}$, $\beta_{h}$ cannot be independently identified from the current dataset. Accordingly, these baseline parameters were adopted from a previously validated HTV-SiR hygrothermal model [3]. That model was developed from multiple hygrothermal conditions and further validated against field-aged insulators in a representative hot–humid region, thus providing the closest validated hygrothermal reference for the 50 °C/85% RH considered in this study. For a similar HTV-SiR material system, Ref. [3] also reported a TGA turning temperature of approximately 350 °C and apparent activation energies ranging from 0.29283 to 0.30249 eV under different hygrothermal conditions, suggesting relatively consistent reaction mechanisms within that hygrothermal regime. By adopting the reported values $R_{a}=0.302\;\mathrm{eV}$, $\beta_{h}=16.08$, the unknown parameter vector that remains to be estimated is reduced to $\theta =\{\alpha ,\rho ,m,\sigma^{2},\beta_{e},\beta_{m},\mu ,\delta \}$.

Existing approaches typically assume a specific probability distribution for X(0) and estimate the parameters of stochastic degradation models using maximum likelihood estimation (MLE) or the expectation–maximization (EM) algorithm [26]. However, due to the limited sample size in our experiments, it is impractical to reliably infer the underlying distributional form of X(0). To circumvent this limitation, we employ the generalized method of moments (GMMs) for parameter estimation. For the degradation model in Equation (7), the following moment conditions hold identically:

(8) $$E[X(t)]=E[X(0)]-k\cdot t^{\rho }$$

(9) $$\mathrm{Var}[X(t)]=\mathrm{Var}[X(0)]+\delta^{2}t$$

where E[·] and Var[·] denote the mean and variance functions, with m=E[X(0)], $\sigma^{2}=Var[X(0)]$. Equations (8) and (9) indicate that the mean–time relationship depends solely on parameters m and the aging coefficient k, whereas the variance–time relationship is governed exclusively by parameters $\sigma^{2}$ and $\delta^{2}$. Leveraging this decoupling, we implement a two-stage GMM estimation procedure, as detailed below:

Stage 1: Estimation of m, ρ, $\sigma^{2}$, δ and $k_{i}$ under each aging condition. Since the initial tensile strength X(0) is unaffected by aging, unaged specimens are first tested to obtain m and $\sigma^{2}$. Subsequently, for each aging condition (E,M), all available mean–time and variance–time data are utilized. $\rho ,k_{i},\delta^{2}$ are estimated via least-squares fitting by minimizing the discrepancy between the model-predicted and experimentally observed moments:

(10) $$(\rho ,k_{i})=\arg\underset{k,\rho }{\min}\sum_{i=1}\sum_{j=1}{\left[{\bar{X}}_{i}(t_{j})-(m+k_{i}\cdot t_{j}^{\rho })\right]}^{2}$$

(11) $$\delta^{2}=\arg\underset{\delta }{\min}\sum_{i=1}\sum_{j=1}{\left[{s_{i}}^{2}(t_{j})-(\sigma^{2}+\delta^{2}t_{j})\right]}^{2}$$

where j indexes the inspection times, i indexes the aging conditions, ${\bar{X}}_{i}(t_{j})$ and ${s_{i}}^{2}(t_{j})$ denote the sample mean and variance at time $t_{j}$ under the *i*-th aging condition.

Stage 2: Estimation of $\alpha ,\beta_{e},\beta_{m},\mu$. Taking the natural logarithm of Equation (6):

(12) $$\ln k=A+\ln\alpha -\beta_{e}\ln E-\beta_{m}\ln M-\frac{\mu }{H\cdot T\cdot E\cdot M}$$

where $A=-\frac{R_{a}}{K_{B}T}-\frac{\beta_{h}}{H}$ is a constant. By substituting the first-stage estimates $k_{i}$ and the corresponding aging conditions (E,M) into Equation (12), the remaining parameters are estimated via least-squares regression:

(13) $$\left(\alpha ,\beta_{e},\beta_{m},\mu \right)=\mathrm{argmin}\Sigma \left[\ln k-\left(A+\ln\alpha -\beta_{e}\ln E\right.\right.{\left.\left.-\beta_{m}\ln M-\mu /(H\cdot T\cdot E\cdot M)\right)\right]}^{2}$$

The proposed two-stage GMM procedure relies solely on the first- and second-order moment structure of the degradation model and does not require any strong parametric assumptions about the underlying distribution of X(0). Consequently, it offers greater robustness compared to MLE and EM methods, particularly when the sample size is limited.

This study employs the Levenberg–Marquardt algorithm [26] to solve the least-squares problem, ensuring robust convergence. The computational results are presented in Table 4. The physical meanings of the variables can be further interpreted in terms of the degradation mechanisms of HTV-SiR. Specifically, the initial state distribution $X(0)\sim D(m,\sigma^{2})$ characterizes the average initial tensile strength and its specimen-to-specimen dispersion, thus reflecting the initial integrity of the polymer network and the variability caused by manufacturing heterogeneity and pre-existing defects. The chemical reaction factor k represents the effective drift rate of tensile strength degradation under coupled stresses, while the time exponent ρ captures the nonlinearity of the degradation process, which is consistent with the experimentally observed transition from early oxidative crosslinking to later chain scission. The diffusion term in the Wiener process characterizes the stochastic fluctuations induced by microstructural heterogeneity, local defect evolution, environmental perturbations, and measurement noise. In the acceleration factor, α defines the baseline aging scale, $R_{a}$ is an apparent activation-related parameter describing the thermal sensitivity of the dominant aging reactions, $\beta_{h}$ quantifies the contribution of humidity-assisted degradation, $\mu_{H,T}$ reflects the interaction between temperature and humidity, $\beta_{e}$ and $\beta_{m}$ quantify the sensitivities of degradation to electric field strength and tensile strain, respectively, and μ characterizes the synergistic coupling among the four stress factors. Therefore, these parameters are not merely fitting coefficients but compact descriptors of the dominant degradation mechanisms and their uncertainty propagation in HTV-SiR.

### 4.4. Model Validity Verification and Life Prediction

#### 4.4.1. Model Validity Verification

Based on the parameter estimates obtained via the two-stage GMM procedure, the proposed multi-stress coupled stochastic degradation model was fully determined. The validity of the model was verified from two dimensions: the envelopment of degradation trajectories and the accuracy of the mean prediction.

**Figure 15:**
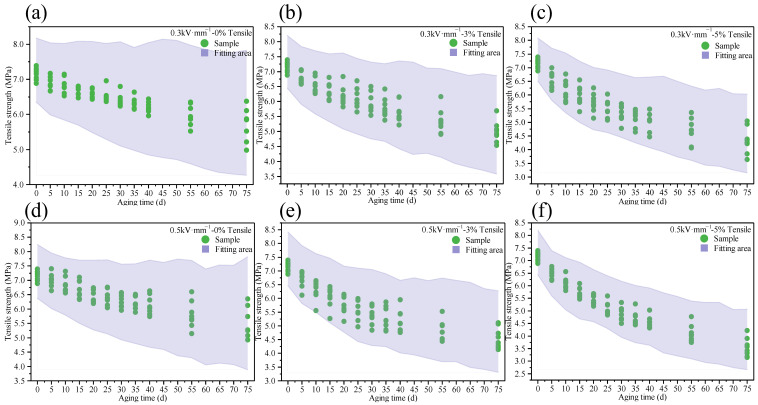
Comparison between Monte Carlo simulated degradation trajectories and experimental observations: (**a**) 0.3 kV·mm^−1^-0% tensile. (**b**) 0.3 kV·mm^−1^-3% tensile. (**c**) 0.3 kV·mm^−1^-5% tensile. (**d**) 0.5 kV·mm^−1^-0% tensile. (**e**) 0.5 kV·mm^−1^-3% tensile. (**f**) 0.5 kV·mm^−1^-5% tensile.

To evaluate the model’s capability in capturing data dispersion, 10,000 virtual samples were generated and compared with the real data points, as shown in Figure 15. The results indicate that the experimental observations at each aging stage fall entirely within the probabilistic envelope of the simulated trajectories, demonstrating the high physical fidelity of the stochastic model.

To further verify the consistency between the predicted mean and the actual degradation level, the mean relative errors $MRE_{stoch,\exp}$ between the stochastic model’s predicted mean and the experimental sample mean were calculated according to Equation (14). These results were compared with the $MRE_{stoch,\det}$ of the deterministic model’s fitted values, as summarized in Table 5. The comparison reveals that the proposed stochastic model is capable of accurately tracking the average aging trend of the material.

(14) $$MRE=\frac{1}{N}\sum_{j=1}^{N}RE_{j}=\frac{1}{N}\sum_{j=1}^{N}\left|\frac{{\hat{X}}_{\mathrm{stoch}\;}\left(t_{j}\right)-\bar{X}\left(t_{j}\right)}{\bar{X}\left(t_{j}\right)}\right|\times 100\%$$

where N denotes the total number of sampling points, ${\hat{X}}_{\mathrm{stoch}\;}(t_{j})$ represents the mean value predicted by the stochastic model at $t=t_{j}$, and $\bar{X}(t_{j})$ signifies the experimental sample mean (or the deterministic model’s fitted mean) at $t=t_{j}$.

In summary, while accurately reproducing the average aging trend, the proposed stochastic model also explicitly characterizes the statistical uncertainty inherent in the degradation process. Compared with traditional deterministic models that provide only point estimates, the stochastic framework offers a more informative and reliability-oriented basis for lifetime prediction of silicone rubber materials under complex multi-stress service conditions.

#### 4.4.2. Life Prediction

To evaluate the service life of HTV-SiR materials under multi-stress coupling conditions, the First Passage Time (FPT) theory was introduced. By defining $D_{f}$ as the failure threshold, the lifetime T was characterized as the time when the degradation process first crosses this critical boundary:

(15) $$T=\inf\left\{t:X(t)\leq D_{f}\right\}$$

By performing multiple stochastic simulations via the Monte Carlo method, the empirical distribution of the lifetime T could be obtained. Subsequently, the survival function S(t) is constructed as:

(16) S(t)=P(T>t)

where S(t) represents the probability that the material remains functional at time t. Based on the survival function, the lifetime quantile $T_{q}$ is defined as the time point satisfying the following condition:

(17) $$P\left(T\leq T_{q}\right)=q$$

According to the DL/T 376-2010 standard [27], the tensile strength of HTV-SiR for composite insulators must not fall below 4.0 MPa. Based on Equation (15)–(17), the predicted lifetime quantiles for HTV-SiR under coupled hygrothermal and electromechanical stresses could be calculated. Taking the “0.5 kV·mm^−1^-5% tensile” condition as an example, during the accelerated aging tests, three specimens failed (approx. 37.5%) after 55 d, and seven specimens failed (approx. 87.5%) after 75 d. By setting the quantiles to q=0.375 and q=0.875, the stochastic degradation model yields predicted lifetimes of 58.2 d and 77 d, respectively. These results demonstrated high consistency with the experimental observations, verifying the accuracy of the proposed lifetime prediction model.

To further assess the model’s practical applicability, the ±800 kV UHVDC transmission project in the Guangzhou region is analyzed. The regional climate is characterized by a mean temperature of 26.9 °C and a mean relative humidity of 78% RH. Within the rated mechanical load range, the stress–strain relationship of the composite insulator core rod can be approximated by Hooke’s Law:

(18) F=K·ε

where F denotes the mechanical load, ε represents the deformation, and K is the stiffness coefficient.

**Figure 16:**
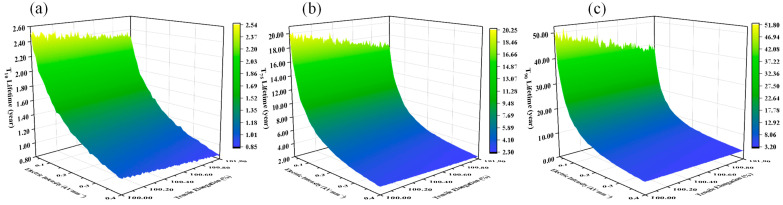
Full-lifetime degradation models at typical quantiles under actual operating strain. (**a**) T_10_ lifetime model. (**b**) T_75_ lifetime model. (**c**) T_90_ lifetime model.

Based on the experimental results in Section 2.2.1, the stiffness coefficient is determined to be $K=6.69\times 10^{6}$. According to [28], the mechanical load on the composite tension insulators for this specific transmission line ranges from 180 kN to 473.9 kN, which corresponds to an actual operating tensile strain of 0.2–0.7%. Under these engineering conditions, the full-lifetime models were established for three typical quantiles ($T_{10}$, $T_{75}$, $T_{90}$), as illustrated in Figure 16.

**Figure 17:**
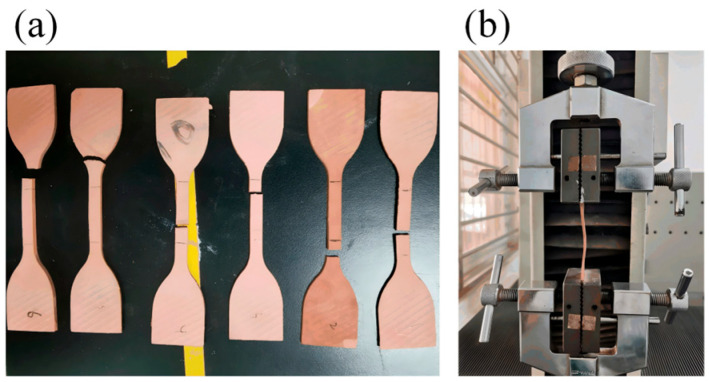
On-site aging tensile testing of insulators. (**a**) Dumbbell-shaped specimen. (**b**) Tensile test.

To validate the proposed model using field data, shed materials were sampled from four composite tension insulators aged for 10 years. For each string, 30 dumbbell-shaped specimens were prepared for tensile testing, as shown in Figure 17. A total of 92 specimens exhibited tensile strengths below the 4.0 MPa threshold, corresponding to an empirical failure ratio of approximately 76.67%, which is close to $T_{75}$.

By setting the operational parameters to an average electric field of 0.09 kV·mm^−1^ and a tensile strain of 0.2%, the predicted $T_{10}$, $T_{75}$ and $T_{90}$ lifetime are 1.77, 9.08, and 17.90 years. In practice, aging conditions cannot remain at their peak levels year-round. Therefore, this theoretical prediction is broadly consistent with the field observation, thereby confirming the practical reliability of the proposed model in real-world grid applications.

## 5. Conclusions

In this study, the aging behavior of HTV-SiR under coupled electro–thermos–hygro–mechanical stresses was systematically investigated, and a stochastic lifetime prediction model incorporating a generalized Eyring acceleration relation was established for destructive tensile test data. The main conclusions are as follows:

1. Under coupled multi-stress aging, HTV-SiR undergoes a transition from early oxidative crosslinking to later main-chain scission. Accordingly, the crosslink density increases initially and then tends to stabilize, whereas tensile stress suppresses the further development of the crosslinked network. Under the same electric field, increasing tensile strain significantly reduces the peak increment in crosslink density.
2. The tensile strength decreases monotonically with aging time, while the hardness continuously increases. The DC breakdown strength exhibits a “rise-then-fall” trend due to the competition between crosslinking and scission. Meanwhile, the relative permittivity and DC conductivity increase continuously because of the accumulation of polar groups and low-molecular-weight degradation products.
3. Traditional deterministic models are not sufficient for life prediction based on one-shot destructive data such as tensile testing because they cannot explicitly account for specimen-to-specimen variability and degradation randomness. By combining a stochastic degradation process with a generalized Eyring acceleration relation, the proposed model enables reliability-oriented lifetime evaluation under coupled stresses. At 0.09 kV·mm^−1^ and 0.2% tensile strain, the predicted lifetimes corresponding to failure probabilities of 10%, 75%, and 90% are 1.77, 9.08, and 17.90 years, respectively, and the applicability of the model is supported by field-aged samples.

A limitation of the present study is that only one temperature–humidity combination (50 °C/85% RH) was experimentally investigated, and the thermal decomposition temperature and thermal-kinetic parameters of the present HTV-SiR were not directly characterized. Future work will therefore extend the test matrix to multiple temperature–humidity levels, combine TGA/DTG-based thermal analysis with coupled electro–mechanical aging tests, and further incorporate cyclic loading, contamination effects, and larger field datasets to improve the transferability, physical interpretability, and engineering applicability of the proposed lifetime model.

## Figures and Tables

**Table 1:** Constituents and basic properties of the HTV-SiR specimen.

Constituent	Range (wt%)	Parameter	Range
Methyl vinyl silicone rubber	42.80~44.00	Hardness (Shore A)	64.00–69.00
Silica	10.30~11.60	Tensile strength (MPa)	7.00–7.50
Aluminum hydroxide	45.30~46.30	Tear strength (kN·m^−1^)	14.00–14.30
		Tensile strain at break (%)	100.00–174.00

**Table 2:** Aging test conditions.

Stress Factor	Level
Temperature	50 °C
Humidity	85.0% RH
Electric intensity	0.30 kV·mm^−1^, 0.50 kV·mm^−1^
Tensile strain	0%, 3%, 5%
Aging time	5, 10, 15, 20, 25, 30, 35, 40, 55, 75 d

**Table 3:** Fitted equations under various aging conditions.

Aging Conditions	Fitting Equation	Coefficient of Determination ($R^{2}$)
0.3 kV·mm^−1^-0% tensile	X(t)=7.01572exp(−0.0029t)	0.981
0.3 kV·mm^−1^-3% tensile	X(t)=6.88217exp(−0.0046t)	0.973
0.3 kV·mm^−1^-5% tensile	X(t)=6.6408exp(−0.0064t)	0.942
0.5 kV·mm^−1^-0% tensile	X(t)=7.0750exp(−0.0036t)	0.979
0.5 kV·mm^−1^-3% tensile	X(t)=6.6992exp(−0.0059t)	0.930
0.5 kV·mm^−1^-5% tensile	X(t)=6.6410exp(−0.0090t)	0.966

**Table 4:** Parameter estimation results.

Parameter	Estimation Value	Parameter	Estimation Value
α	$6.03\times e^{-31}$	$\beta_{e}$	−0.533
ρ	0.68	$\beta_{m}$	−16.687
m	1.98	μ	8.672
$\sigma^{2}$	$0.003^{2}$	δ	0.004

**Table 5:** Comparison of MRE among experimental data, deterministic model, and stochastic model.

Aging Conditions	$MRE_{stoch,\exp}$ (%)	$MRE_{stoch,\det}$ (%)
0.3 kV·mm^−1^-0% tensile	0.64	0.66
0.3 kV·mm^−1^-3% tensile	0.61	1.26
0.3 kV·mm^−1^-5% tensile	2.39	2.69
0.5 kV·mm^−1^-0% tensile	1.95	0.90
0.5 kV·mm^−1^-3% tensile	1.86	2.97
0.5 kV·mm^−1^-5% tensile	2.20	3.08

## Data Availability

The original contributions presented in this study are included in the article. Further inquiries can be directed to the corresponding authors.

## Abbreviations

The following abbreviations are used in this manuscript:

HTV-SiR	High-Temperature Vulcanized Silicone Rubber
PDMS	Polydimethylsiloxane
LMW	Low-Molecular-Weight
ATH	Aluminum Trihydrate/Aluminum Hydroxide
TTS	Time–Temperature Superposition
MLE	Maximum Likelihood Estimation
EM	Expectation–Maximization algorithm
GMM	Generalized Method of Moments
MRE	Mean Relative Error
